# Serum Lactate and Mortality in Emergency Department Patients with Cancer

**DOI:** 10.5811/westjem.2018.6.37295

**Published:** 2018-07-26

**Authors:** Steven A. Maher, M’hamed Temkit, Matthew R. Buras, Ryan Y. McLemore, Rebecca K. Butler, Yasmynn Chowdhury, Christopher A. Lipinski, Stephen J. Traub

**Affiliations:** * Mayo Clinic Arizona, Department of Emergency Medicine, Phoenix, Arizona; † Mayo Clinic, College of Medicine, Rochester, Minnesota; ‡ Mayo Clinic Arizona, Division of Health Sciences Research, Phoenix, Arizona

## Abstract

**Introduction:**

Patients with malignancy represent a particular challenge for the emergency department (ED) given their higher acuity, longer ED length of stay, and higher admission rate. It is unknown if patients with malignancies and hyperlactatemia are at increased risk of mortality. If serum lactic acid could improve detection of at-risk patients with cancer, it would be useful in risk stratification. There is also little evidence that “alarm” values of serum lactate (such as >/=4 mmol/L) are appropriate for the population of patients with cancer.

**Methods:**

This was a continuous retrospective cohort study of approximately two years (2012–2014) at a single, tertiary hospital ED; 5,440 patients had serum lactic acid measurements performed in the ED. Of the 5,440 patients in whom lactate was drawn, 1,837 were cancer patients, and 3,603 were non-cancer patients. Cumulative unadjusted mortality (determined by hospital records and an external death tracking system) was recorded at one day, three days, seven days, and 30 days. We used logistic regression to examine the risk of mortality 30 days after the ED visit after adjusting for confounders.

**Results:**

In an unadjusted analysis, we found no statistically significant difference in the mortality of cancer vs. non-cancer patients at one day and three days. Significant differences in mortality were found at seven days (at lactate levels of <2 and 4+) and at 30 days (at all lactate levels) based on cancer status. After adjusting for age, gender, and acuity level, 30-day mortality rates were significantly higher at all levels of lactic acid (<2, 2–4, 4+) for patients with malignancy.

**Conclusion:**

When compared with non-cancer patients, cancer patients with elevated ED lactic acid levels had an increased risk of mortality at virtually all levels and time intervals we measured, although these differences only reached statistical significance in later time intervals (Day 7 and Day 30). Our results suggest that previous work in which lactate “cutoffs” are used to risk-stratify patients with respect to outcomes may be insufficiently sensitive for patients with cancer. Relatively low serum lactate levels may serve as a marker for serious illness in oncologic patients who present to the ED.

## INTRODUCTION

Cancer is a growing healthcare problem, and it is the second leading cause of death in the United States (U.S.). 1 There were 1,665,540 new cancer cases and 585,720 cancer deaths in the U.S. in 2014, 2 and this number is expected to rise by 70% over the next 20 years. 3 Importantly, cancer diagnoses tend to increase exponentially with age, and the fastest growing segment of the U.S. population is adults over age 65. 4 Cancer, therefore, is poised to play an increasingly prominent role in emergency medicine (EM).

Previous work suggests that emergency department (ED) serum lactate has an association with mortality in a broad array of ED patients including those with suspected sepsis or meeting sepsis criteria, hemorrhagic shock, focal ischemic conditions, metabolic derangement, congestive heart failure, and low-flow states. 5–11 We sought to determine the role of lactate in predicting mortality specifically in ED patients with cancer. Vital signs and biological markers are typically used to risk stratify acutely ill patients in the ED. Patients with malignancies may have subtle presentations of acute disease, owing either to comorbid conditions or an immunosuppressed state from chemotherapy. If serum lactate could improve detection of at-risk patients with cancer, it would be useful in risk stratification. Furthermore, there is little evidence that “alarm” values of serum lactate (such as >/= 4 millimoles per liter [mmol/L]) are appropriate for the population of patients with cancer.

We report the association of serum lactate and unadjusted mortality at one, three, seven, and 30 days on patients with and without malignancy, and adjusted mortality (based on age, sex, and Emergency Severity Index [ESI] score) at 30 days. We analyzed these data in categorical fashion using lactate intervals of <2 mmol/L (normal), 2 to <4 mmol/L, or >\= 4 mmol/L.

## MATERIALS AND METHODS

This was a continuous, retrospective cohort study of all ED visits from December 1, 2012, to August 31, 2014. The Mayo Clinic Arizona ED is a single, tertiary, teaching hospital located in Phoenix, Arizona. During the study period, annual ED volume was approximately 28,000 with substantial seasonal variation. The ED is staffed year-round by board-certified emergency physicians. The admission rate during the study period was approximately 30%. The Mayo Clinic institutional review board process approved this study and waived the requirement of informed consent.

All visits during the study period in which a serum lactate was drawn in the ED prior to patient disposition were eligible for analysis. We did not include admitted patients whose first serum lactate was drawn after admission (even if that blood draw occurred in the ED) or patients who did not have a serum lactate drawn. The decision to order a serum lactate was left to the discretion of the treating physician. At our facility, a serum lactate is typically ordered in patients with suspected sepsis or meeting sepsis criteria, hemorrhagic shock, focal ischemic conditions, metabolic derangement, and other low-flow states. During the study period, blood was collected by laboratory phlebotomists soon after ordering and immediately taken to the laboratory for quantitative analysis; the use of phlebotomists (vs. nurses) to draw blood samples did not produce significant delays in sample procurement. There were no changes during the study period in how laboratory personnel collected and processed samples.

From our electronic medical record (EMR) (Cerner^®^, Kansas City, MO), we extracted patient age, presence of cancer, race, acuity, and gender; ESI score; and serum lactate level. We defined age in integral years on the date of registration. The presence of cancer was determined by a field present on the past medical history portion of the nursing ED treatment record. This record was completed on each patient upon presentation to the ED. We defined gender and race based on patient declaration. We assessed race as white vs. non-white; we aggregated non-white responses due to the low number of non-white patients in our sample. We assessed ESI score in binary (1/2 vs. 3/4/5) fashion. Serum lactate was measured in mmol/L using a Roche Cobas 6000 serum-based assay (Indianapolis, IN) located in our central laboratory. We did not use point-of-care lactate testing during the study period. One author (RB) was responsible for all data abstraction from the EMR.

**Population Health Research Capsule**

What do we already know about this issue?

Previous work suggests that emergency department (ED) serum lactate has an association with mortality in a broad array of ED patients but has not been well studied in cancer patients.

What was the research question?

What is the role of lactate in predicting mortality specifically in ED patients with cancer?

What was the major finding of the study?

Cancer patients with elevated ED lactic acid levels had an increased risk of mortality at day 7 and day 30.

How does this improve population health?

It alerts clinicians to the fact that relatively low serum lactate levels may serve as a marker for serious illness in oncologic patients who present to the ED.

Cumulative mortality (determined by hospital records and an external death tracking system) was recorded at one day, three days, seven days, and 30 days.

We divided the cohort into patients who had active cancer or a history of cancer vs. those who did not. The primary outcome was patient mortality, and the primary comparison was cancer patients vs. non-cancer patients. Cumulative mortality was noted at one, three, seven, and 30 days. We report unadjusted mortality by lactate level at one, three, seven, and 30 days, as well as adjusted mortality (adjusted via multivariable logistic regression in a model that included age, gender, ESI score, and race) at 30 days.

The limited number of events at one, three, and seven days precluded a meaningful regression analysis in these groups. The descriptive statistics consist of frequencies and proportions for categorical variables, and mean and standard deviation for continuous variables. We used the Pearson chi-square to test for univariate associations between categorical values and the Wilcoxon rank-sum for univariate comparisons of numerical variables between two groups where appropriate (Table 1). Statistical Analysis Software (SAS) (Version 9.4, SAS Institute, Cary, NC) was used for all analysis.

## RESULTS

There were 47,136 ED visits during the study period; 5,440 had a serum lactate measurement performed in the ED and were eligible for analysis. Of the 5,440 patients in whom lactate was drawn, 1,837 were cancer patients, and 3,603 were non-cancer patients. The baseline characteristics of the 5,440 study visits are shown in Table 1.

### Emergency Severity Index

Patients with cancer were more likely to be older, male, and have a more acute ED presentation (as evidenced by lower ESI scores). Unadjusted mortality results are presented in Table 2.

The information from Table 2 is represented graphically in Figure. When stratified by lactate level (<2 mmol/L, 2 to <4 mmol/L and >/= 4 mmol/L), there were no statistically significant differences in mortality at one day or three days at any level; differences in the <2 mmol/L and the >/= 4 mmol/L levels, but not in the 2 to <4 mmol/L level at seven days; and differences at every lactate level at 30 days. In all cases in which there were differences, mortality was higher in the cancer group.

Adjusted mortality results at 30 days are presented in Table 3. In the adjusted 30-day analysis, patients with cancer had a greater mortality than patients without cancer at every lactate level. A low number of mortality events precluded a determination of adjusted mortality results at the one-, three- or seven-day time points.

## DISCUSSION

Serum lactate is a useful biomarker in EM. Elevations in lactate correlate with mortality in a broad array of ED patients, including those with suspected sepsis or meeting sepsis criteria, hemorrhagic shock, focal ischemic conditions, metabolic derangement, congestive heart failure, and low-flow states. 5–11 Specifically, a serum lactate ≥4 has been shown to be associated with significant mortality, regardless of the etiology of elevation. 12–14

Patients with cancer are unique in many ways, in that they may be malnourished, chronically ill, and immunosuppressed. It is not surprising, therefore, that in-hospital septic cancer patients have a higher mortality, longer hospital length of stay, and higher total costs than septic non-cancer patients; 15, 16 and they also have a higher mortality from invasive pneumococcal infections. 17 Our findings of elevated lactate levels correlating with mortality in patients with and without malignancy are consistent with previous work in this area. Of note, however, many of the previous investigations of lactate and mortality have specifically focused on infection.^8^,^13^,^14^,^18^–^26^ Our work does not attempt to categorize patients with respect to the reason why a lactate was determined, but rather seeks to understand the degree to which serum lactate can be used as a prognostic tool in patients with cancer.

In an unadjusted analysis, we found a consistent increase in mortality at almost all lactate levels in patients with cancer as compared to patients without cancer; however, at the earlier time points (day 1 and day 3) these findings were not statistically significant. By day 7, there was a statistically significant difference in mortality at two lactate levels (0–2 and >/= 4) that did not reach statistical significance for the lactate level of 2–4. At 30 days, there was a statistically significant difference at all levels. An analysis controlling for age, gender, and acuity confirmed the statistically significant difference in mortality at 30 days.

Our work builds upon that of others to establish that patients with malignancy represent a high-risk group in the ED. Oncology patients who present to the ED with sepsis and bacteremia have a higher 72-hour mortality, higher in-hospital mortality, and higher 28-day mortality than non-cancer patients. 27 In addition to the burden of chronic illness, cancer patients are more likely to be receiving immunosuppressive chemotherapy, which may predispose cancer patients to greater morbidity and mortality. This may be particularly true in disease states (such as infection or sepsis) that would predispose a physician to obtain a serum lactate level.

Our results suggest that previous work in which lactate “cutoffs” are used to risk-stratify patients with respect to outcomes may be insufficiently sensitive for patients with cancer. For example, at 30 days the unadjusted mortality rate of cancer patients with a lactate of 2–4 was 12.83%, almost double the 6.83% mortality of non-cancer patients with a level of 2–4. At 30 days the unadjusted mortality of patients with a lactate of four or greater in cancer vs. non-cancer was 39.24% vs. 21.77%. These findings also were significant at 30 days after adjusting for age, gender, and acuity. The 30-day adjusted mortality rates of cancer patients at the 2–4 and 4+ lactic acid levels were almost double that of non-cancer patients (9.25% vs. 5.73% and 27.4% and 16.1%, respectively).

We note that in the unique population of cancer patients, the prognostic value of serum lactate levels may serve another purpose. It may help guide end-of-life decisions in patients who are terminally ill. Such decisions are often difficult, and are particularly difficult to make in a fast-paced environment such as the ED. While the extrapolation of our work for this purpose is limited by the fact that we did not stratify cancer patients into those who were near end of life vs. those who were not, our findings may be of use in such cases, or at least provide a framework for further research and discussion in this area.

We found elevated rates of mortality at essentially every lactate level and time interval in cancer patients as compared with non-cancer patients, although many of those differences did not reach statistical significance in the earlier timeframes. While we acknowledge that there are limitations in our methodology, we nonetheless believe that our work suggests that relatively low serum lactate levels may serve as a marker for serious illness in oncologic patients who present to the ED. This may require a shift in clinical mindset, as many clinicians typically consider an “alarm” value of lactic acid to be at or above four. Since even the adjusted 30-day mortality of cancer patients nearly doubles at the 2–4 lactic acid level and then almost doubles again at the 4+ level, clinicians may need to pay special attention to this especially vulnerable population of cancer patients in the 2–4 lactic acid level range. Further prospective research is needed.

## LIMITATIONS

Our data were from a single site at which the percentage of ED visits for patients with cancer (~20%) is quite likely higher than that of most EDs. Both of these factors limited generalizability. Additionally, we obtained mortality data from an internal database and external death tracking system, but did not attempt to contact patients (such as by phone); thus, we may have failed to identify deaths that occurred but were not recorded in either of our databases. We had no reason to believe, however, that missed events would have occurred in one group more than another.

We treated cancer categorically. We did not differentiate patients by type of malignancy, did not determine if patients in the cancer group had an active malignancy or simply a history of cancer, and did not record the presence or absence of active chemotherapy or radiation. We believe that our decision to view cancer patients in the aggregate allowed for a high-level analysis of this group, but we concede that there are myriad details regarding lactate levels in each of the abovementioned subgroups that we cannot know.

We lack data necessary to characterize our findings of lactic acidosis as Type A (due to hypoperfusion) or Type B (related to mitochondrial dysfunction) or their relation to the Warburg effect. These limitations are particularly important given our focus on cancer patients, as Type B lactic acidosis is a rare, often-fatal complication in some patients with lymphoma, leukemia, and solid malignancies. In the Warburg effect, cancer cells produce additional energy through increased oxygen-dependent glycolysis followed by lactic acid fermentation with secretion of lactate.

We analyzed only mortality. There may have been significant differences in morbidity between the two groups (such as admission to a monitored bed, endotracheal intubation, or need for invasive monitoring), but we did not have data to ascertain this. And because our data are retrospective, we were able to determine correlation but not causation. While our data showed a strong correlation between patients with cancer and lactic acid elevation, a prospective trial would suggest causation.

Our relatively low event rate at earlier (one-day and three-day) time points limits our ability to draw conclusions in those timeframes. The fact that the higher mortality seen in cancer patients vs. non-cancer patients at almost every lactate level did not reach statistical significance may have been due to a relatively small sample size, not necessarily a lack of effect.

Our data suffer significantly from selection bias. Emergency physicians ordered a serum lactate at their discretion, without set criteria. For example, approximately 34% of our lactate samples were drawn in the 20% of our ED visits made by patients with cancer; this may have reflected the fact that patients with cancer were more systematically ill, or may have reflected an age-related bias in ordering lactate levels. We did not attempt to ascertain the clinical reasoning behind the decision to order a serum lactate; anecdotally, it appeared that providers ordered serum lactate preferentially in infected patients or those who were seriously ill. We have no reason to believe, however, that this pattern of ordering lactate is different to that of other EDs, or that ordering behavior was different in cancer vs. non-cancer patients.

As we lacked a robust mechanism to clearly delineate ED visits that were or were not related to other ED visits for the same patient, we treated each ED visit as an independent event. This is a limitation, as one ED visit may affect another, particularly if the visits are temporally proximate. Finally, our data may not adequately control for severity of illness. Although we incorporated ESI scores into our model, ESI may not fully reflect and differentiate patients with respect to illness severity.

## CONCLUSION

In summary, cancer patients with elevated ED lactic acid levels had an increased risk of mortality at virtually all lactate levels and time intervals that we measured, although these differences only reached statistical significance in later time intervals (day 7 and day 30). These results suggest that previous work in which lactate “cutoffs” were used to risk-stratify patients with respect to outcomes may be insufficiently sensitive for patients with cancer. Relatively low serum lactate levels may serve as a marker for serious illness in oncologic patients who present to the ED.

## Figures and Tables

**Figure:**
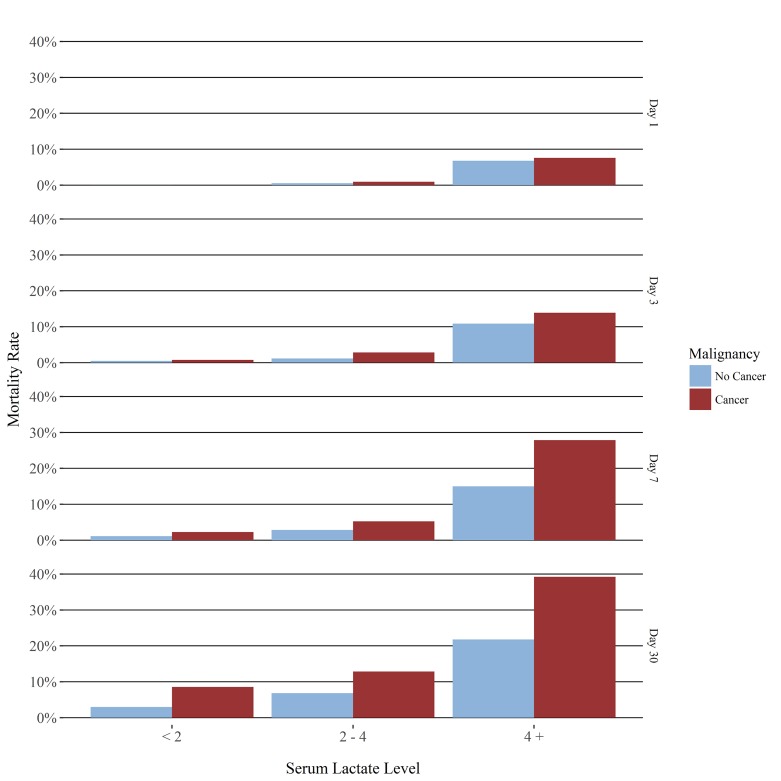
Mortality rate at day 1, 7, 30: cancer vs. no cancer.

**Table 1:** Patient information.

	Cancer n=1837	No cancer n=3603	Difference	95% CI of difference	P value
Age (years)	69.8(14.0)	62.4(19.2)	7.4	6.5–8.3	< 0.01
Gender					< 0.01
Female	845 (46.0%)	1933 (53.6%)			
Male	992 (54.0%)	1670 (46.4%)	7.7%	5.7% to 9.6%	
Race					< 0.01
Caucasian	1737 (94.6%)	3315 (92.0%)			
Other	100 (5.4%)	288 (8.0%)	−2.5%	−3.5% to −1.6%	
ESI					< 0.01
1 or 2	655 (35.9%)	1101 (30.6%)			
3, 4, or 5	1172 (64.1%)	2502 (69.4%)	−5.3%	−7.2% to −3.4%	
Lactate (mmol/L)					0.45
0–2	1337 (72.8%)	2690 (74.4%)	−1.6%	−4.1 to 0.8%	
2–4	421 (22.9%)	776 (21.5%)	1.4%	−0.1 to 3.7%	
4+	79 (4.2%)	147 (4.1%)	0.1%	−0.1% to 1.3%	

*CI*, confidence interval; *ESI*, Emergency Severity Index.

**Table 2:** Unadjusted mortality by cancer and lactate levels. Data presented as absolute mortality (mortality rate).

Lactate	Cancer n=1837	No cancer n=3603	Difference	95% CI of difference
1 day				
<2	0(0%)	1(0.04%)	−0.04%	NA
2–4	4(0.95%)	4(0.52%)	0.48%	−0.62% to 1.49%
4+	6(7.59%)	10(6.80%)	0.79%	−6.33% to 7.91%
3 days				
<2	10(0.75%)	13(0.49%)	0.26%	−0.27% to 0.79%
2–4	12(2.85%)	9(1.16%)	1.69%	−0.06% to 3.45%
4+	11(13.92%)	16(10.88%)	3.04%	−6.10% to 12.18%
7 days				
<2	30(2.24%)	30(1.12%)	1.12%	0.24% to 2.01%
2–4	22(5.23%)	22(2.84%)	2.39%	−0.03% to 4.82%
4+	22(27.85%)	22(14.97%)	12.88%	1.44% to 24.33%
30 days				
<2*	114(8.53%)*	80(2.99%)*	5.54%*	3.91% to 7.17%*
2–4	54(12.83%)	53(6.83%)	6.00%	2.34% to 9.65%
4+*	31(39.24%)*	32(21.77%)*	17.47%*	4.81% to 30.1%*

*CI*, confidence interval.

* Statistically significant differences.

**Table 3:** Adjusted mortality at 30 days. Mortality rates after adjusting for age, gender, and acuity level (ESI).

Lactate	Cancer n=1837	No cancer n=3603	Difference	95% CI of difference
1 day				
<2*	7.31%*	3.19%*	4.12%*	2.19%–4.85%*
2–4*	9.25%*	5.73%*	3.52%*	0.57%–5.49%*
4+*	27.4%*	16.1%*	11.3%*	4.06%–29.66%*

*CI*, confidence interval; *ESI*, Emergency Severity Index.

* Statistically significant differences.
